# Supplementary material for: The Basis of Tolerance Mechanism to Metsulfuron-Methyl in Roegneria kamoji (Triticeae: Poaceae)
Source: Plants (Basel). 2021 Sep 1;10(9):1823. doi: 10.3390/plants10091823 (PMC8466435; doi:10.3390/plants10091823)
Supplement: Supplementary file 1 [file plants-10-01823-s001.zip › plants-1346885-supplementary.pdf]

Supplementary figures

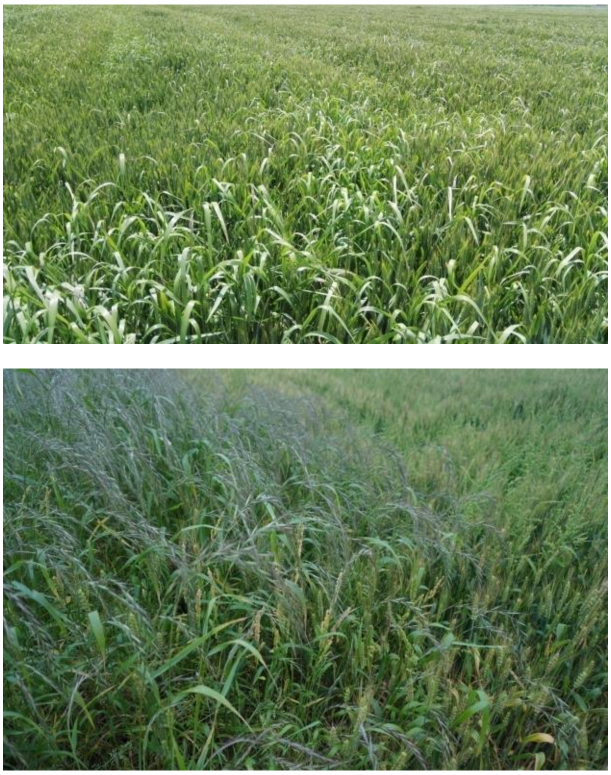

Supplemental Figure S1: *R. kamoji* infested wheat fields: Jingzhou, 2017 (upper); Haiyan, 2017 (lower).

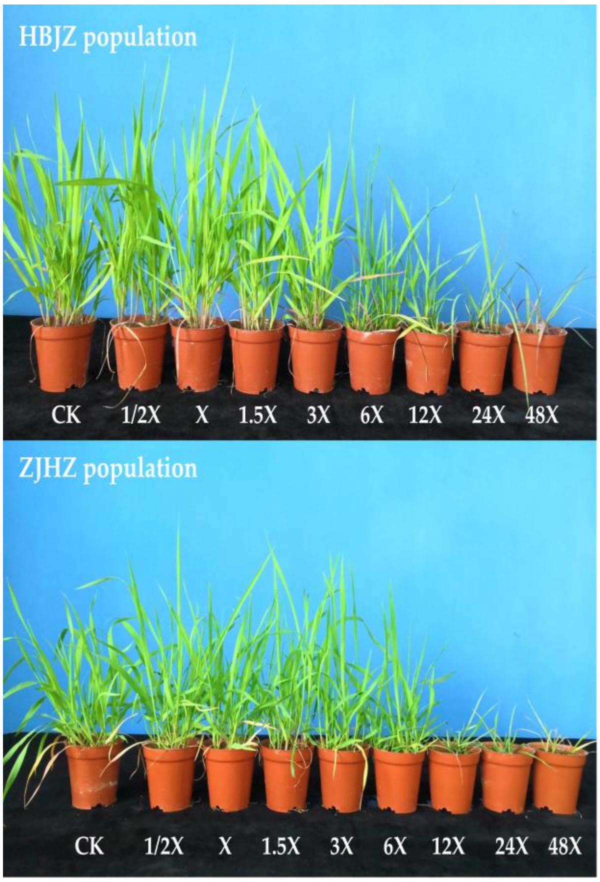

Supplemental Figure S2: Photographs of HBJZ and ZJHZ *R. kamoji* populations 28 days after treated with different doses of metsulfuron-methyl (X=7.5 g ai ha^{-1})

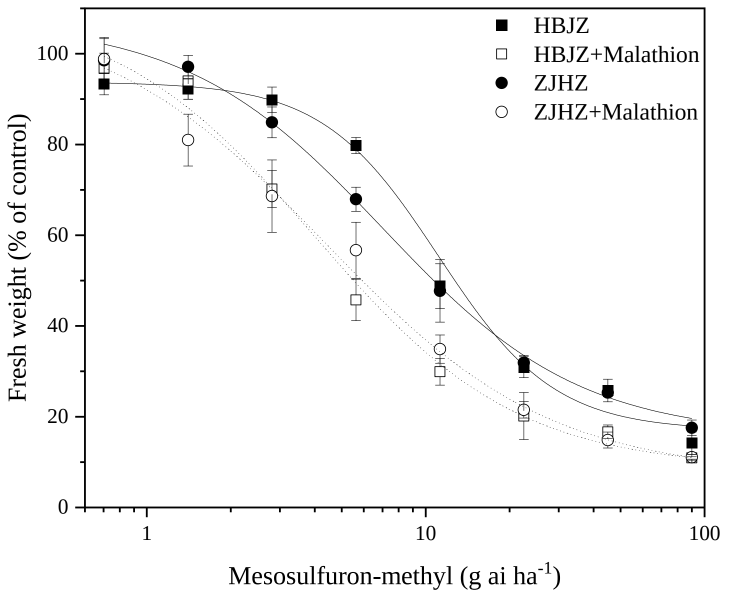

Supplemental Figure S3: Dose response curves for the fresh weight (% of control) of the HBJZ and ZJHZ *R. kamoji* populations treated with a range of mesosulfuron-methyl doses with (1000 g ai ha^{-1}) or without malathion pretreatment. Each point is the mean ± SE of the two experiments each containing four replicates.
